# Mechanical properties of dense mycelium-bound composites under accelerated tropical weathering conditions

**DOI:** 10.1038/s41598-021-01598-4

**Published:** 2021-11-11

**Authors:** Xin Ying Chan, Nazanin Saeidi, Alireza Javadian, Dirk E. Hebel, Manoj Gupta

**Affiliations:** 1 Department of Mechanical Engineering, National University of Singapore (NUS), Singapore, 117575 Singapore; 2 Sustainable Construction, Faculty of Architecture, Karlsruhe Institute of Technology (KIT), 76131 Karlsruhe, Germany

**Keywords:** Civil engineering, Mechanical engineering, Bioinspired materials

## Abstract

Mycelium, as the root of fungi, is composed of filamentous strands of fine hyphae that bind discrete substrate particles into a block material. With advanced processing, dense mycelium-bound composites (DMCs) resembling commercial particleboards can be formed. However, their mechanical properties and performance under the working conditions of particleboards are unknown. Here, we show how weathering conditions affect the DMC stress and elastic modulus. DMC was made using *Ganoderma lucidum* mycelium grown on a substrate of sawdust and empty fruit bunch. The DMC was then subjected to weathering under tropical conditions over 35 days and tested under flexural, tensile, and compressive loading with reference to international standards. After exposure to specified weathering conditions, the maximum stress in flexure, tension, and compression decreased substantially. The addition of a protective coating improved the resistance of DMC to weathering conditions; however, the difference between coated and uncoated samples was only found to be statistically significant in tensile strength.

## Introduction

Particleboards, also known as chipboards, are a class of engineered wood products commonly used in the construction and furniture industry that are in high demand. They come in a variety of thicknesses and sizes and are made by chemically binding and mechanically pressing fine wood shavings into desired finishing. Wood shavings are obtained mainly from scrap wood residues from other wood applications^1^; hence, particleboards are often considered to be an environmentally sustainable alternative to hardwood. However, the growing market for particleboards still propels the need for deforestation^2^. This has resulted in negative environmental impacts, as forests are finite resources that need years to regenerate. Hence, it is important to reduce the dependency on fresh wood and wood-based products to achieve environmental sustainability and reduce greenhouse gas (GHG) emissions.

Another problem in the production of particleboards lies in the adhesive used to bind the wood particles together. For decades ago, particleboards were bonded primarily using urea–formaldehyde (UF)-based adhesives^3,4^, as they are cheap and bind well easily^5^. In Europe, 90–92% of the adhesives used for particleboards are UF based^6^. However, UF adhesives are known to produce formaldehyde emissions, which can be detrimental to human health. Emissions cause major discomfort to humans when inhaled at high concentrations and could even cause cancer^7,8^. Furthermore, UF adhesives, together with other petroleum-based resins, rely on nonrenewable resources and are not environmentally friendly or sustainable^9^. These adhesives are also nonbiodegradable and can increase waste production at the end of the life of products^10^.

In response to the problem of UF binders being harmful to human health and the environment, many research studies have focused on developing bioadhesives^7,9–12^. These include adhesives made from various biopolymers, such as cellulose, starch, and latex^7^. However, such adhesives remain highly flawed substitutes for traditional adhesives, as studies on such adhesives have reported poor bonding strength, poor water resistance, longer production times, and a higher cost of production compared to traditional adhesives^7,9,10^.

Li et al. created an adhesive made from tannin, a plant-based biopolymer^13^. When applied as a wood adhesive, it showed good water resistance and binding strengths. However, the adhesive had a short storage period of only 2 h. Beyond which, the adhesive strength decreases drastically. This can create logistical problems if the adhesive is applied commercially, as it cannot be stored for a longer duration. Hence, it is not a viable option for commercial particleboard production.

In another study, Yamada et al. created an adhesive using a chitosan biopolymer, which had high water resistance and binding strength. However, it was problematic to synthesize the adhesive because it had a high viscosity^14^. High viscosity in adhesives is usually not favoured in particleboard production, as it has adverse effects on adhesive penetration. Wood adhesive penetration is essential for the bonding mechanism between wood fibres and the adhesive matrix, which determines the mechanical properties of particleboards^15–18^. This is the major reason for the poor performance of many bioadhesives when used in wood composite bonding^9^. This process is defined by the liquid adhesive flowing into the wood particles through pores on the wood surface^19,20^. Mechanical bonds are formed with this process, with proper penetration being achieved by the adhesives penetrating the microfeatures of the material, such as cracks in the cell walls of the wood particles^21^. The high viscosity of adhesives causes insufficient penetration, which reduces the effectively bonded areas of the material, hence reducing the overall material strength^21,22^.

On the other hand, mycelium-based composites do not depend on wood adhesive penetration for strength. Mycelium is the root part of fungi and is composed of filamentous strands of fine white hyphae^23^. It grows with increasing density in its substrate, hence binding the substrate together. The structure and strength of the hyphae are given by chitin in the cell walls^24,25^. Chitin is a hard, inelastic linear polymer^26^ bonded by hydrogen bonds, which gives it rigidity along its chain^27^. This bonding allows chitin to possess superior tensile strength, even higher than that of carbon fibre and steel^28^.

When a substrate is inoculated with fungi, the mycelium grows by digesting the substrate for nutrients^27^. In this process, mycelium spreads through the substrate with a networked structure, which becomes denser in the substrate with continued growth^29^. This means that, for instance, when mycelium grows on a sawdust substrate, the hyphae penetrate the wood particles and bind them together. Eventually, with enough coverage of the mycelium, the sawdust particles are bonded into a single block of material.

Many factors affect the growth of mycelia, such as growth medium, incubation time, temperature, moisture level, and acidity^28^. The effects of these factors vary for different fungal species. Generally, fungi grow well with 5–14 days of incubation^30^ on a slightly acidic substrate of pH 4–6^31^.

*Ganoderma lucidum (G. lucidum)* is a common fungus due to its commercial value. It is commercially grown in farms for its fruiting body, which is commonly known as Lingzhi in traditional Chinese medicine^32^. Its optimal growth pH is approximately 6.0, and it grows best at temperatures of approximately 30 °C^33,34^. *G. lucidum* is easy to cultivate, as it grows readily on most plant-based substrates^35^. *G. lucidum* is also classified as a basidiomycete, which means that it can rapidly digest lignin, changing the chemical structure of lignin into lignin-based radicals^36^. With a sufficient supply of oxygen, these radicals can form cross-links and act as an adhesive^36^. Under the right conditions, growth of mycelium occurs at exponential rates^37^; hence, it is able to bond to its substrate in a period as short as 5–7 days^38^.

The other component of the DMC developed in this study is the empty fruit bunch (EFB). EFB is the leftover fruit bunch fibre from the extraction of oil from palm fruits^39^. It is the byproduct of palm oil production in Southeast Asia. In 2012 alone, 43.2 and 76.9 million tons of empty fruit bunches were generated in Malaysia and Indonesia, respectively^40,41^. Its abundance as a waste byproduct of the palm oil industry has led to its recognition as an underutilized resource ^42^. Unfortunately, when EFB is discarded without proper processing, it releases greenhouse gases into the environment^43^. Studies have been initiated to make better use of EFB as a natural resource, with some using it as a substrate for mushroom cultivation^41–44^. In some studies, particleboards were made from EFB using UF adhesives as the binder^45^.

In a study by Suzuki et al., a binder-less board was created using oil palm fronds^46^. In that study, fronds were dried and hot-pressed into a board at 125–150 °C, with no additional adhesives. The boards had mechanical properties that satisfied the Japanese industrial standards. It was revealed that lignin, a biopolymer, contributed to the board’s self-binding properties. EFB also contains lignin^47^. Given its self-binding properties, any lignin undigested by mycelium could also contribute to bonding within the mycelium-based composite. Therefore, this study will incorporate EFB into mycelium-based composites as part of efforts to reduce the wastage of natural resources.

Composites comprise at least 2 distinct phases, usually a matrix and a fibre^48^. Fibre is used to improve the mechanical properties or increase the volume of the composite^49^. Particleboards are also considered a class of composite materials made from sawdust and adhesives. Due to their wood-based composition, their properties are significantly affected by environmental factors such as moisture and temperature^50^. Particleboard degrades quickly within 1–2 weeks of weathering, such that even if they are painted, the paint would not be able to form a good bond on the surface^51^. Hence, particleboards are made mainly for indoor use^1^.

Given the environmental impacts of traditional particleboards, this paper proposes dense mycelium-based composites (DMCs) as a potential alternative to traditional particleboards. Specifically, the combination of EFB and mycelium could potentially replace a material with both high demand and negative environmental impacts with one that upcycles waste products and could even eliminate the use of harmful chemicals. Mycelium-based composites are highly versatile and have been developed for different applications, such as 3D printing^52^, packaging materials^53^, home appliances^54^, textiles^55,56^, and construction^57–59^. In these applications, the material is subjected to indoor weathering, which determines the lifespan and functional integrity of the material. However, there have been very few studies on the longevity of materials under practical weathering conditions^57^. This is important for ensuring that the application of mycelium-based material would not fail prematurely due to weathering conditions experienced in tropical environments. Hence, this paper will focus on the production of an EFB-based DMC and evaluate its performance after being subjected to tropical weathering conditions.

## Methods

### Materials

The mycelium mother culture of *G. lucidum* (M 9720) was purchased from Mycelia bvba (Nevele, Belgium). The mycelium mother culture was then maintained on potato dextrose agar (PDA) slopes, incubated at 28 °C for up to 7 days and stored at 4 °C for subsequent subculturing. PDA media was made from the following components in grams per litre: potato extract, 4 g; dextrose, 20 g; and agar, 15 g. Individual components were purchased from Sigma Aldrich, St. Louis, Mo.

EFB fibres were collected from the Heng Huat Group in Malaysia, and sawdust from *Albizia chinensis*, a common tropical plant, was collected from a San Ho timber workshop in Singapore. Mycelium growing substrates were supplemented with 10% (W/W) wheat bran (Bob’s Red Mill, Product of USA) for additional nutrition and 2% (W/W) calcium carbonate (CaCO_3_) (LushGro, Singapore) to adjust the mixture pH level. Some of the DMC samples were coated with an oil-based coating from Osmo Holz und Color (Warendorf, Germany) for weathering tests. Verification of the mycelium species and plant fibres used in this research was carried out with companies that handed over the respective materials for the research. Necessary documents were provided to the Singapore custom office to ensure complying with Singapore laws in terms of imported live specimens.

### Sample preparation

EFB fibres and sawdust were oven-dried upon delivery at 70 °C for 2 h to reach a humidity of ~ 10%. This step was mainly carried out to avoid contamination during the storage of fibres before usage. Prior to usage, dry EFB fibres were mixed with water to reach a humidity of 60%, which is a favourable condition for the growth of mycelium^64^. Water and dry EFB were mixed at a ratio of 5:4 by weight. The fibres were then packed in polypropylene (pp) bags, sterilized at 120 °C for at least 20 min and then cooled to room temperature.

Oven-dried sawdust was sieved using a Fritsch vibratory sieve shaker (Fritsch, Germany) to remove particles larger than 2 mm in diameter. Afterwards, sawdust, wheat bran, and CaCO_3_ were dry mixed with W/W percentages of 88%, 10% and 2%, respectively, and the mixture was then mixed with water at proportions of 40% and 60%. The mixture was subsequently packed in 2 L pp bags, hot sealed, sterilized at 120 °C for 60 min, and cooled to room temperature before inoculation with mycelium grain spawn. Each bag approximately weighed 1 kg.

The sterilized EFB and colonized sawdust bags were mixed and gently packed into moulds with dimensions, as shown in Supplementary Figure 1a and 1b. The moulds were designed according to the size of the pressing machine (180 cm in length and 30 cm in width). EFB and sawdust were mixed in proportions of 4:3 by volume. Five and a half kilograms of mixture was used for the type A mould (23.1 L) while 4 kg is used for type B (16.5 L). This produces a DMC block with an average wet density of approximately 239 kg/m^3^.

### DMC fabrication

Organic wheat grains, wheat bran, and CaCO_3_ were dry mixed with weight percentages (wt%) of 88%, 10%, and 2%, respectively. Afterwards, the mixture was mixed with 20 wt% water, sealed in 2 L pp bags, sterilized at 120 °C for 60 min, and subsequently cooled to room temperature (RT) prior to inoculation with *G. lucidum* mycelium on PDA plates. Using a scalpel, mycelium was cut into 5 mm squares, and the whole plate was mixed with a sterilized grain bag. Each grain spawn bag was approximately 1.5 kg. The bag was then closed with a reusable ring and cotton ball as a filter and left in an incubation room at 26–28 °C with 70–80% humidity for 2 weeks to develop mycelium grain spawn for further usage.

Sawdust, wheat bran, and CaCO_3_ were dry mixed with weight percentages (wt%) of 88%, 10%, and 2%, respectively. The mixture was then mixed with 60 wt% water, packed in 2 L pp bags, hot sealed and sterilized at 120 °C for 60 min and cooled to RT before inoculation with mycelium grain spawn. Each bag weighed approximately 1 kg. Afterwards, each bag was mixed with 1 g of fully colonized mycelium spawn, closed, and incubated, as explained in the previous section. After 2 weeks of incubation, the bags were fully colonized with mycelium. Supplementary Figure 2a and 2b show the sawdust bags before and after colonization by *G. lucidum* mycelium.

After successful colonization of sawdust bags, they were mixed with sterilized EFB and packed into the final composite moulds, as explained in the previous section. The moulds were then left for incubation under similar conditions previously explained. After 4–5 days, when the mycelium network was observed to have spread evenly on the external surfaces of the substrate, the mould was removed. The mycelium was left to grow for another 3–4 more days until the mycelium fully covered the surface of the substrate. A slightly longer inoculation time results in a smoother surface on the mycelium^65^. Shakir et al. also found that a longer inoculation time did not affect the mechanical properties of the resulting DMC board^66^. Hence, the mycelium blocks used were grown for 7–9 days to achieve similarity in terms of mycelium coverage on the external surface of all the blocks.

### Post-processing

Upon completion of the growth cycle, mycelium-based composites were kept in an oven at a temperature of 70 °C for another 2 days until their weight stabilized and did not change overnight. The final density of the mycelium blocks was in the range of 120–130 kg/m^3^ after drying. Supplementary Figure 3 shows the mycelium blocks after drying.

Dried mycelium-based composite blocks were arranged using 2 pieces of type A blocks at the side and 1 piece of type B block in the middle and placed in a hot press compression moulding machine with a width of 30 cm and length of 190 cm, as shown in Supplementary Figure 4. Afterwards, the boards were pressed at a temperature of 120 °C, with the pressing pressure on the machine set to 20 MPa for a duration of 50 min. The final composite board was cured for an additional 24 h in an oven at a temperature of 50 °C after removal from the hot press to ensure gradual adjustment to ambient temperature. This method helps to reduce material defects caused by a sudden change in moisture and temperature content^50^. The resultant density of the specimens cut from the board was approximately 954 kg/m^3^.

### Sample preparation and characterization

**Figure 1:**
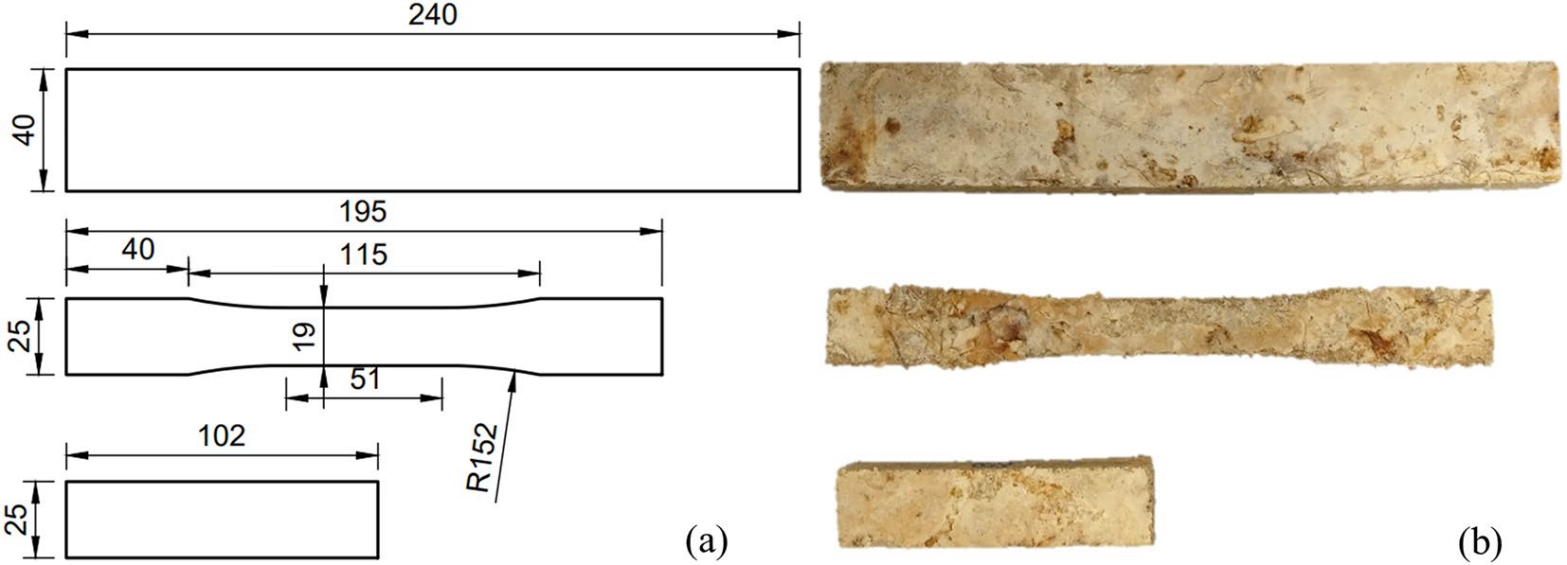
Testing specimens: (**a**) dimensions of testing samples and (**b**) test specimens for flexural, tensile and compression testing (from top to bottom).

The samples were prepared for testing the flexural, tensile, and compressive strength with reference to ASTM D1037^67^. Modifications were made due to limitations in laboratory equipment. The dimensions of the samples are shown in Fig. 1a. After pressing, DMC was cut into samples, as shown in Fig. 1b, using a computerized numerical control (CNC) cutting machine (MultiCAM, USA).

Half of the samples were coated with Osmo oil-based coating according to manufacturer guidelines, and the remainder of the samples were tested with no coating but under similar weathering conditions. The control set was tested before weathering on Day 0, while the remaining samples were kept in a KOMEG weathering chamber (KOMEG, Hong Kong).

The weathering conditions were designed to simulate tropical weathering conditions in Southeast Asia, with the relative humidity controlled at 75(± 15) % and temperature controlled at 27.5(± 2.5) °C. Mycelium-based composites are shown to be resistant to ultraviolet radiation^65^, and the material was not made for applications with direct exposure to sunlight. Hence, ultraviolet light exposure was not included in the weathering parameters. The weathered samples were tested on Days 7, 21, and 35 of exposure. Each set had 3 uncoated samples and 3 coated samples.

The flexural, tensile, and compression tests were conducted using a Shimadzu AIG-100 kN Universal Testing Machine (UTM) (Shimadzu, Japan). From these tests, the maximum stresses in bending ($${R}_{B})$$, compression ($${\mathrm{R}}_{\mathrm{C}})$$ and tension $${(\mathrm{R}}_{\mathrm{T}}$$) and modulus of elasticity in tension ($${\mathrm{E}}_{\mathrm{T}})$$ were recorded. Single-factor ANOVA tests were then conducted on the data obtained to determine the significance of the changes in mechanical properties from Day 0 to Days 7, 21 and 35.

A 4-point flexural test was used to find ${R}_{B}$ and evaluate the flexural properties, as shown in Supplementary Figure 5. The support span was set to 210 mm, and the loading span was set to 70 mm. The cross-head speed of the UTM was 2 mm/min. ${R}_{B}$ was obtained with the equation given in (1)^67–69^. ${P}_{max}$ stands for the maximum load, $\frac{\Delta P}{\Delta y}$ is the slope of the straight portion of the load–deflection curve, and $b, d$ and $L$ represent the specimen width, thickness, and distance between support points, respectively.

1 $${\mathrm{R}}_{\mathrm{B}}=\frac{{\mathrm{P}}_{\mathrm{max}}\mathrm{L}}{{\mathrm{bd}}^{2}}$$

The compressive tests were carried out at a cross-head speed of 0.5 mm/minute, and ${\mathrm{R}}_{\mathrm{C}}$ was recorded. The formula for ${R}_{C}$ is as follows^67^:

2 $${\mathrm{R}}_{\mathrm{C}}=\frac{{\mathrm{P}}_{\mathrm{max}}}{\mathrm{bd}}$$

${\mathrm{R}}_{\mathrm{T}}$ and ${\mathrm{E}}_{\mathrm{T}}$ were obtained from tensile tests to evaluate the properties of the material in tension. A standard tensile test with a cross-head speed of 3 mm/min was carried out. Strain values for obtaining ${\mathrm{E}}_{\mathrm{T}}$ were measured using an Epsilon 3542 Axial Extensometer (Epsilontech, USA). The formulas for ${R}_{T}$ and ${E}_{T}$ are as follows, where ${l}_{g}$ is the gauge length of specimen^67^:

3 $${\mathrm{R}}_{\mathrm{T}}=\frac{{\mathrm{P}}_{\mathrm{max}}}{\mathrm{bd}}$$

4 $${\mathrm{E}}_{\mathrm{T}}=\frac{{\mathrm{l}}_{\mathrm{g}}}{\mathrm{bd}}\frac{\Delta \mathrm{P}}{\Delta \mathrm{y}}$$

As the properties of a material are dependent on its structure^70^, scanning electron microscopy (SEM) was conducted to observe the material structure. For microscopy, the cut cross section of an untested sample of DMC was sputter-coated with gold and observed under a JEOL JSM 6010 tungsten cathode scanning electron microscope at 10 kV.

The effects of weathering were determined using a coating protection (CP) value. This value was derived by taking the difference in maximum stress or elastic modulus of coated and uncoated samples over the coated value. The larger the value is, the greater the effects of the coating. A positive value indicated better strength or rigidity in the coated samples, while a negative value indicated better strength or rigidity in the uncoated samples. The CP is calculated using the formula below, where R is the maximum stress or elastic modulus.

5 $$\mathrm{CP}=\frac{\mathrm{R}\left(\mathrm{coated}\right)-\mathrm{R}\left(\mathrm{uncoated}\right)}{\mathrm{R}\left(\mathrm{uncoated}\right)}\times 100\mathrm{\%}$$

## Results

**Figure 2:**
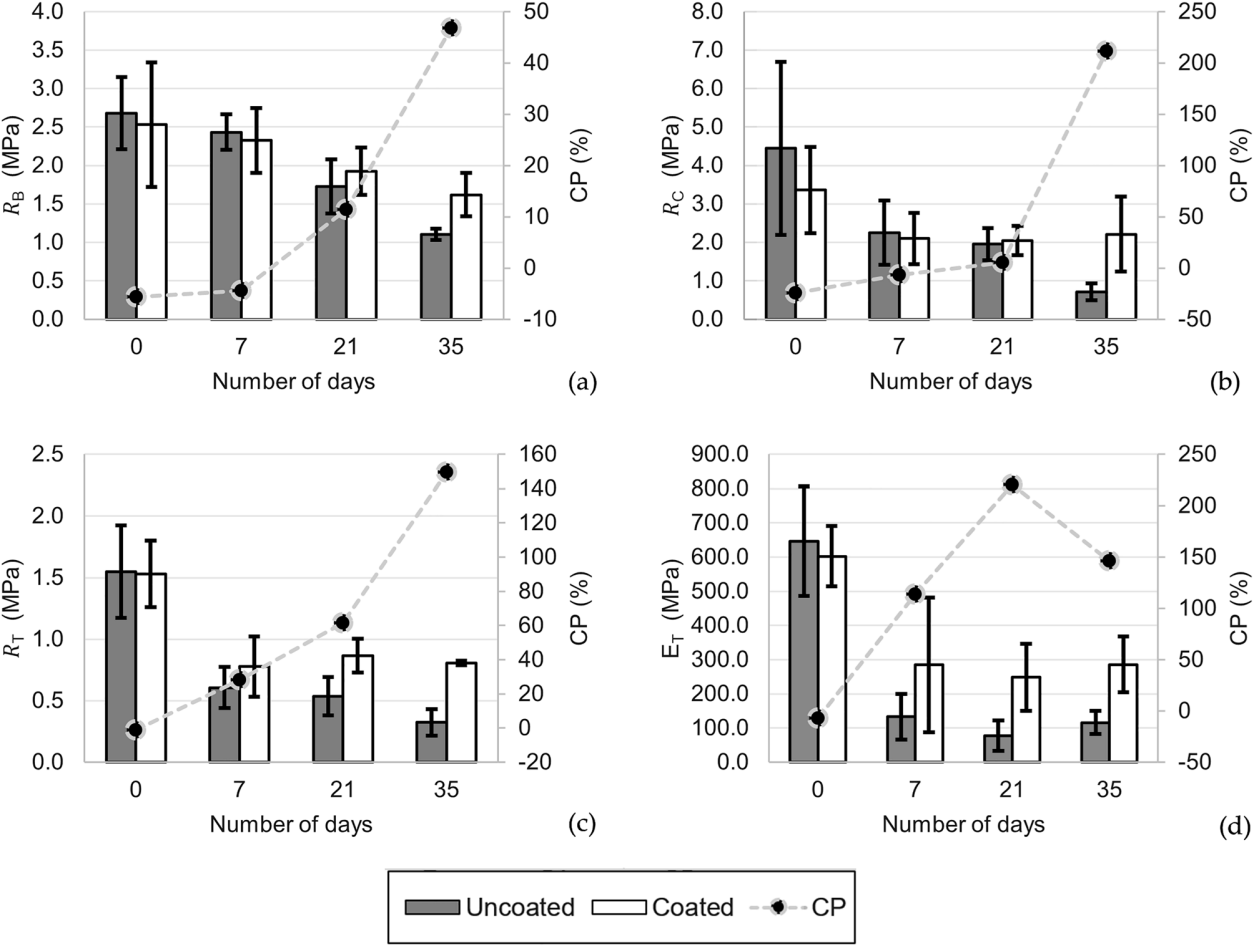
Mechanical test results: (**a**) R_B_ and CP against the number of days exposed to weathering conditions; (**b**) R_C_ and CP against the number of days exposed to weathering conditions; (**c**) R_T_ and CP against the number of days exposed to weathering conditions and (**d**) ET and CP against the number of days exposed to weathering conditions.

After producing DMC materials (Fig. 1) (please see Methods), the samples were cut out and tested for their mechanical properties in batches on Days 0, 7, 21, and 35. The flexural, compression and tensile test results are displayed in Fig. 2 There was a generally decreasing trend in the values of the maximum stresses and elastic modulus of the samples. The difference between the maximum stress and elastic modulus of the uncoated and coated samples on each day was also calculated and plotted as CP values. CP values generally increased over the 35 days. For ${R}_{B}$, ${\mathrm{R}}_{\mathrm{C}}, {\mathrm{R}}_{\mathrm{T}}$ and ${\mathrm{E}}_{\mathrm{T}}$ (see Methods for the definition), a two-factor ANOVA (with replication) was conducted to compare the difference between the mechanical properties of coated and uncoated samples. ANOVA tests were conducted at a 95% confidence interval where *P* < 0.05 showed a statistically significant difference between coated and uncoated samples.

### Flexural

For ${R}_{B}$, as observed in Fig. 2a, there was a notable impact from weathering on both uncoated and coated samples. For uncoated samples, ${R}_{B}$ on Day 0 was 2.68 ± 0.47 MPa, while on Day 35, it decreased by 59.0% to 1.10 ± 0.07 MPa. For coated samples, ${R}_{B}$ on Day 0 was 2.53 ± 0.81 MPa, while on Day 35, it decreased by 36.0% to 1.62 ± 0.28 MPa. Comparing the coated and uncoated samples, the CP values generally increased from − 6% on Day 0 to 47% on Day 35. This means that the coated samples had a higher ${R}_{B}$ than the uncoated samples, and the effect of the coating became more evident as the material was exposed to a more weathering environment. ANOVA revealed that there was no statistically significant difference between the ${R}_{B}$ of coated and uncoated samples under weathering conditions (*P* > 0.05). Supplementary Table 1 shows the raw ${R}_{B}$ data of coated versus uncoated samples and the results of ANOVA performed between the two groups.

Taken together, these results suggest that weathering had a notable impact on the ${R}_{B}$ of DMC. Specifically, when the material is exposed to weathering conditions for a longer period, the flexural properties of DMC start to decrease. Degradation of the material through weathering was also reduced by a protective coating. However, the effect of the protective coating on ${R}_{B}$ was not statistically significant.

### Compressive

As demonstrated in Fig. 2b, there was a substantial decrease in ${R}_{C}$ from Day 0 to Day 35. For uncoated samples, ${R}_{C}$ on Day 0 was 4.44 ± 2.24 MPa, while on Day 35, it decreased by 84.0% to 0.71 ± 0.22 MPa. For coated samples, ${R}_{C}$ on Day 0 was 3.36 ± 1.12 MPa, while by Day 35, it decreased by 34.2% to 2.21 ± 0.98 MPa. CP values increased from − 24% on Day 0 to 211% on Day 35. This shows an increase in the difference between ${R}_{C}$ of coated samples compared to uncoated samples. The results of the ANOVA tests revealed that, there was statistically no significant difference between ${R}_{C}$ of coated and uncoated samples from day 0 to day 35 (*P* > 0.05). Supplementary Table 2 shows the raw ${R}_{c}$ data of coated versus uncoated samples and the results of ANOVA carried out between the two groups.

These results suggest that weathering had a substantial impact on the ${R}_{C}$ of DMC. The longer the material was exposed to weathering conditions, the weaker the material in terms of compressive strength. From the CP values, it can also be seen that the coated samples also had a larger ${R}_{C}$ than the uncoated samples on Days 21 and 35. While there was a difference between the ${R}_{C}$ of coated and uncoated samples, the results of the ANOVA tests indicated that the difference was not statistically significant (*P* > 0.05).

### Tensile

As seen in Fig. 2c, in tension, ${R}_{T}$ was significantly reduced by weathering, while the protective coating had a significant impact on reducing the effects of weathering on ${R}_{T}$. For uncoated samples, ${R}_{T}$ on Day 0 was 1.55 ± 0.37 MPa while on Day 35, it decreased by 79.4% to 0.32 ± 0.11 MPa. For coated samples, ${R}_{T}$ on Day 0 was 1.53 ± 0.27 MPa, while by Day 35, it decreased by 47.1% to 0.81 ± 0.02 MPa. CP values increased from -1% on Day 0 to 149% on Day 35. This means that the difference between ${R}_{T}$ of coated samples and uncoated samples was increasing. The ANOVA tests indicated that there was statistical significance in ${R}_{T}$ between coated and uncoated samples at the end of 35 days (*P* < 0.05). Supplementary Table 3 shows the raw ${R}_{T}$ data of coated versus uncoated samples and the results of ANOVA carried out between the two groups.

As illustrated in Fig. 2d, ${E}_{T}$ was also reduced by weathering. ${E}_{T}$ on Day 0 was 646.8 ± 159.9 MPa, while on Day 35, it decreased by 82.0% to 116.1 ± 34.1 MPa. For coated samples, ${E}_{T}$ on Day 0 was 602.2 ± 87.9 MPa, while by Day 35, it decreased by 52.5% to 286.1 ± 81.7 MPa. CP values increased from − 7% on Day 0 to 146% on Day 35. Based on ANOVA tests, the difference between ${E}_{T}$ of coated and uncoated samples is not significant (*P* > 0.05). Supplementary Table 4 shows the raw ${E}_{T}$ data of coated versus uncoated samples and the results of ANOVA carried out between the two groups.

These results show that weathering had a notable impact on both ${R}_{T}$ and ${E}_{T}$. Although the effects of a coating were not statistically significant with respect to ${E}_{T}$, they were significant with respect to ${R}_{T}$. When DMC is exposed to a longer weathering period, both ${R}_{T}$ and ${E}_{T}$ decrease. This shows that the effects of the coating could be magnified by prolonged weathering.

**Figure 3:**
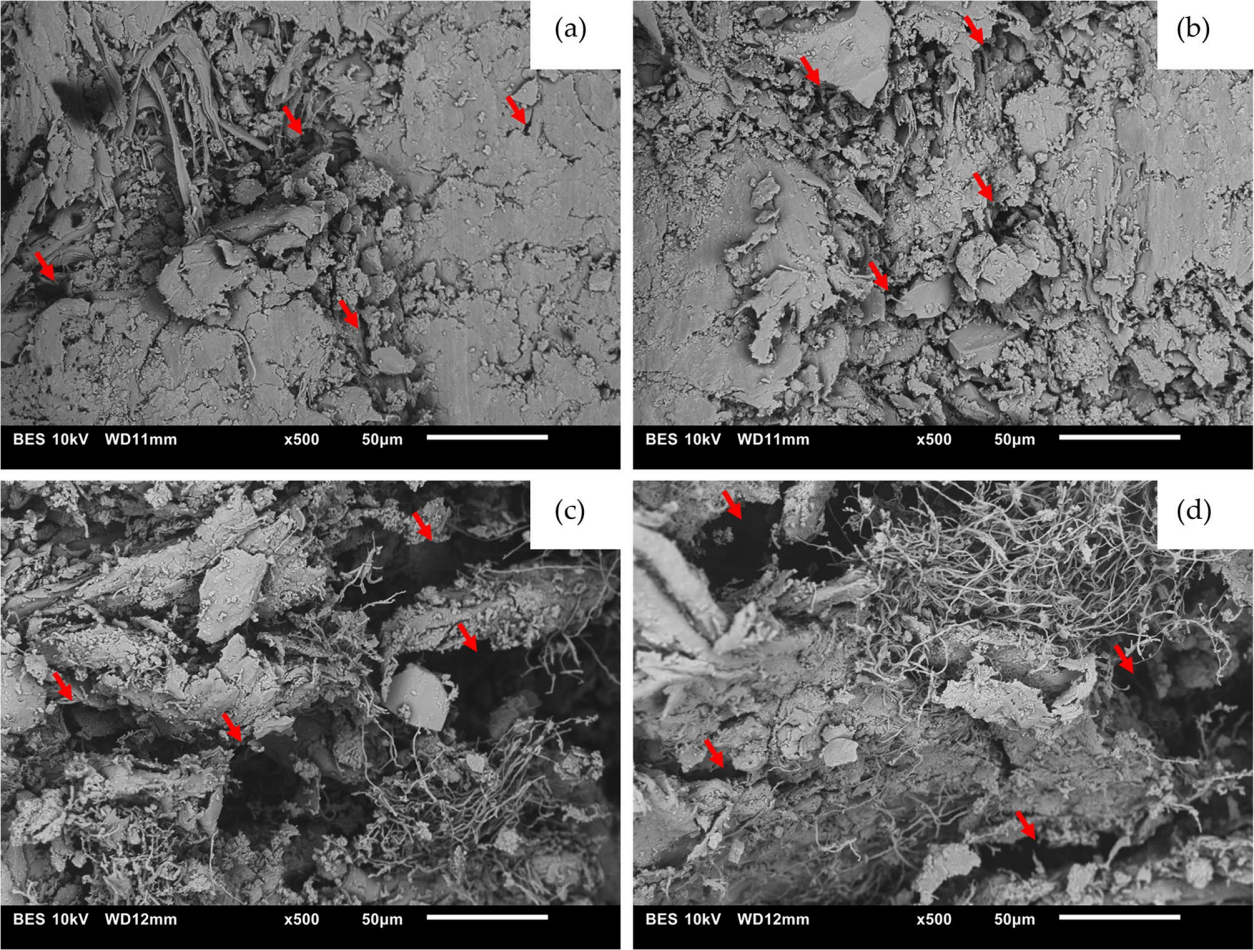
SEM images of different surfaces: (**a**) and (**b**) Surfaces with smoother textures and (**c**) and (**d**), Surfaces with more porous textures. Examples of pores are shown by arrows in the images.

Microscopy images on the surface of the material revealed some inconsistency in terms of the distribution of fibres and porosity. In Fig. 3a and b, the surface is observed to be smoother, with fewer loose fibres present. Figure 3c and d show more visible fibres, as well as a larger number of pores marked by darker areas. This shows significant variation in the microstructure of the material even within a small area. Areas with more pores and other defects are likely to become areas of stress concentration. When these samples were tested, the discontinuity propagates under applied loading and subsequently initiates the failure of the samples below their true maximum stress. Samples of pores are marked by arrows on the images.

## Discussion

As the material was exposed to weathering conditions, there was a general decreasing trend in maximum stress in all 3 modes of loading. This means that the strength of the materials decreases due to the degradation of the wood-based substrate in the material. This is consistent with past studies that have established wood-based material to be sensitive to weathering conditions^50^.

As observed in Fig. 2, at Day 0, compared to coated specimens, some uncoated specimens demonstrate slightly higher ${R}_{B}$, ${\mathrm{R}}_{\mathrm{C}}, {\mathrm{ R}}_{\mathrm{T}}$ and ${\mathrm{E}}_{\mathrm{T}}$ values, which are mainly due to the nonhomogenous structure of the mycelium-bound composite material, as it is naturally grown. However, generally, coated samples showed higher performance over long exposure to environmental conditions.

Although the coating was able to help the material retain some of its strength over the weathering period in all the loading scenarios, it was statistically significant only in preserving ${R}_{T}$. Considering the microscopy results, the porous surface inhibited the coating’s ability to create a perfect seal on the surface of the material. During the coating process, the liquid coating was unable to completely infiltrate the deeper and larger pores, hence allowing moisture to enter the material through surface defects. With a smoother surface, the coating would be able to create a more comprehensive barrier to prevent moisture from entering the material. Therefore, improvement in the consistency of the material surface and microstructure is fundamental for increasing the strength of the material. By doing so, fewer areas of stress concentration leading to crack propagation and material failure would be present, thereby also improving the material strength.

Another way to improve the material strength could be by increasing the heat applied during the pressing process. In addition to chitin’s bonding strength in mycelium, lignin present in EFB and sawdust also determines the mechanical properties of pressed boards. This effect is due to lignin softening and cross-linking reactions^60,61^. Lignin softening occurs at approximately 115 °C^46^. Although the temperature applied during pressing was 120 °C, the thickness of the board may have impeded proper heat transfer to the core of the board within the given pressing time. When insufficient time is given for the material to cure properly, the bonds are only partially formed, hence significantly reducing the strength of the material^62,63^. Lignin reacts to form new cross-links only when exposed to temperatures of approximately 160 °C^61^, which was not the case in this study. Therefore, increasing the pressing temperature to at least 160 °C could improve the strength of the material. However, a higher pressing temperature may also reduce the bonding strength of mycelium hyphae, which needs further investigation.

A mycelium-based composite was successfully developed in this project. The results of this study show that an environmentally friendly alternative to particleboards can be developed from agricultural waste. The material was exposed to weathering conditions over 35 days. On Days 0, 7, 21, and 35, the mechanical properties of the samples were tested using a 4-point flexural test, tensile test, and compression test setup. From the tests, the maximum bending stress, maximum tensile stress, maximum compressive stress, and modulus of elasticity for tension were found. The mechanical test results over the weathering period reveal that weathering significantly reduces the strength and rigidity of the material. A commonly used oil-based coating used in the wood industry applied to the material was able to reduce the degradation of the material under tropical weathering conditions, but its effectiveness was limited in preserving ${R}_{T}$. However, some improvements to the material’s consistency could effectively increase the material strength and resistance to weathering with the help of a protective coating. Therefore, DMC could be a promising material as an environmentally friendly substitute for particleboards if such improvements in material production are made.

## Supplementary Information

Supplementary Information.

## Data Availability

The data that support the findings of this study are available from the corresponding author upon request.
