# Supplementary material for: Mechanical properties of dense mycelium-bound composites under accelerated tropical weathering conditions
Source: Sci Rep. 2021 Nov 11;11:22112. doi: 10.1038/s41598-021-01598-4 (PMC8586338; doi:10.1038/s41598-021-01598-4)
Supplement: Supplementary file 1 — Supplementary Information. [file 41598_2021_1598_MOESM1_ESM.docx]

# Supplementary Note 1

Supplementary Figures


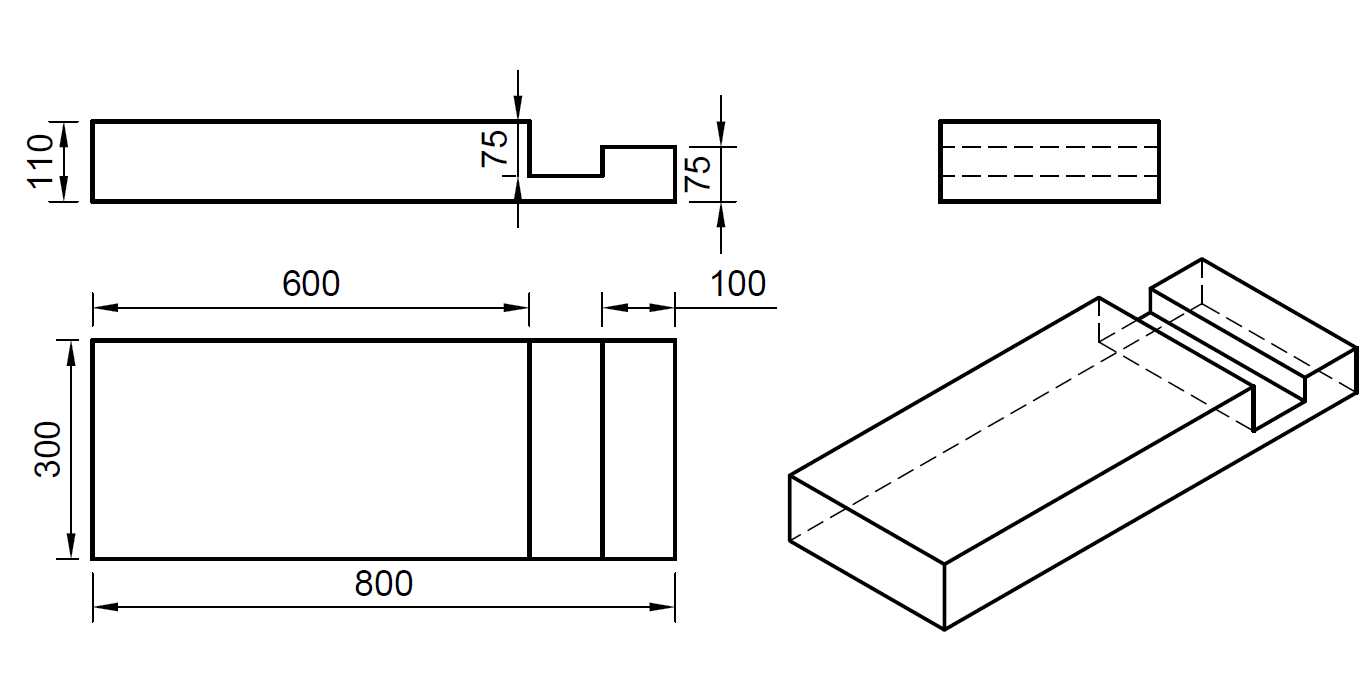


(a)


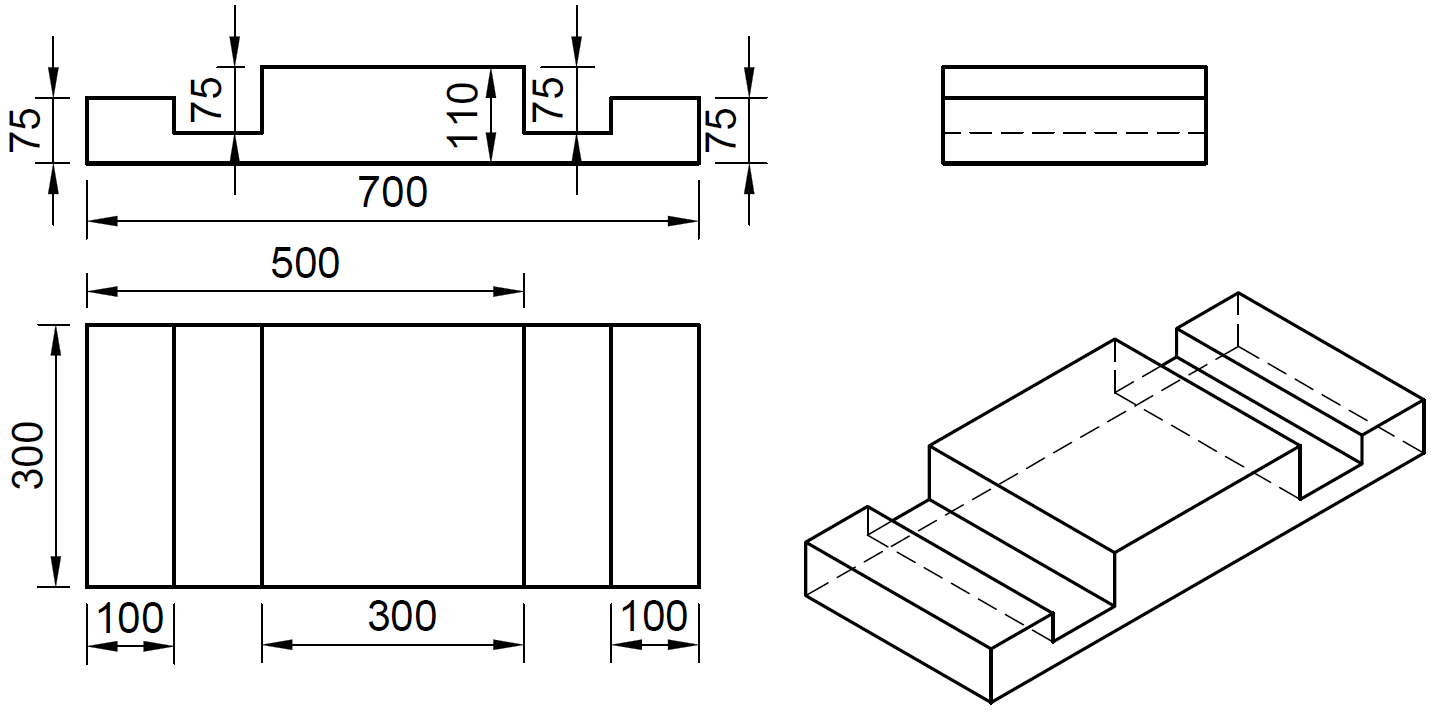


(b)

**Supplementary Fig. 1| Dimensions of moulds. a,** Dimensions of type A mould and **b**, Dimensions of type B mould in mm


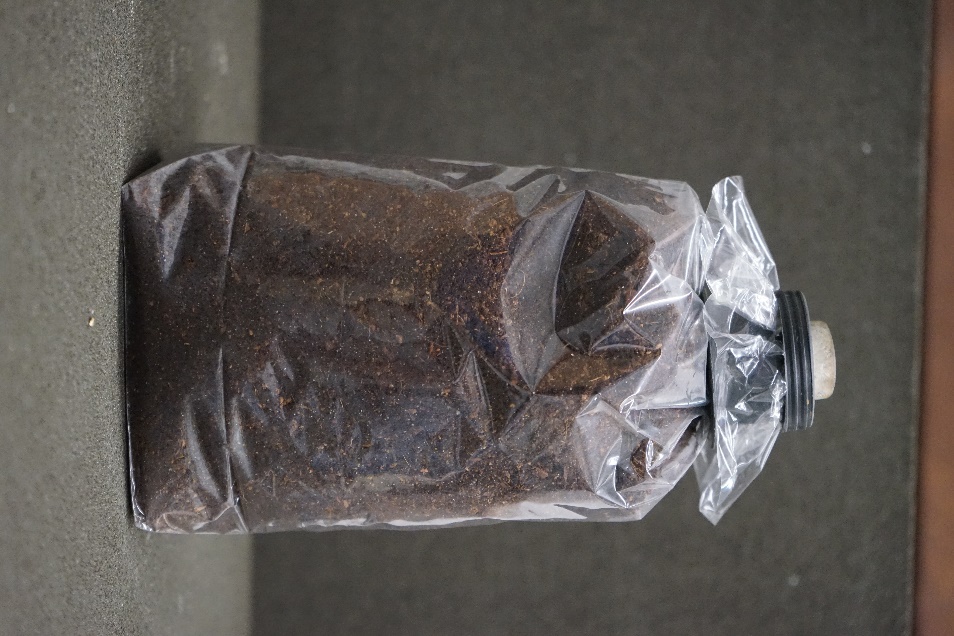

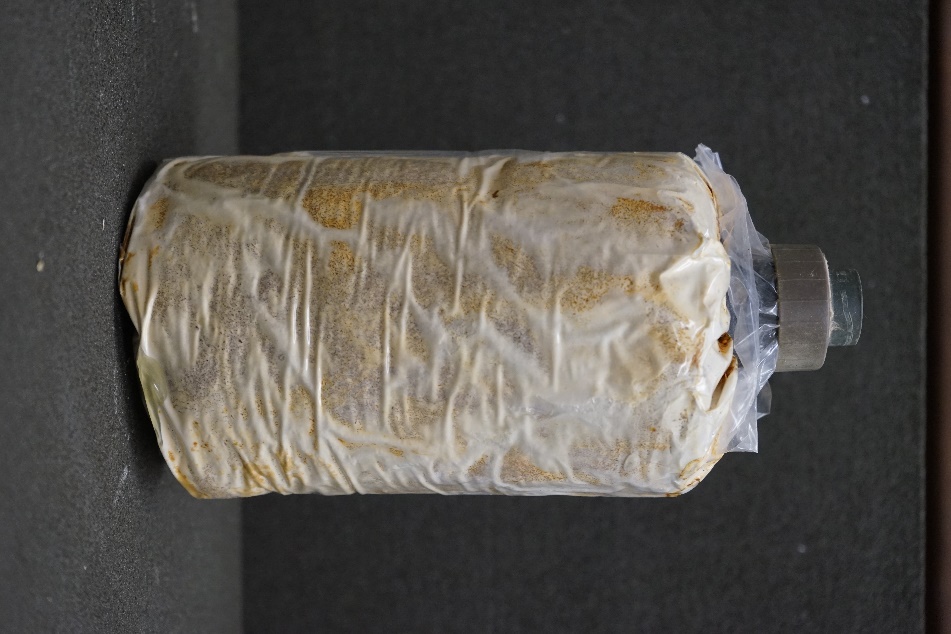


(a)

(b)

**Supplementary Fig. 2|Inoculated sawdust bags**. a, before growth and **b,** after growth


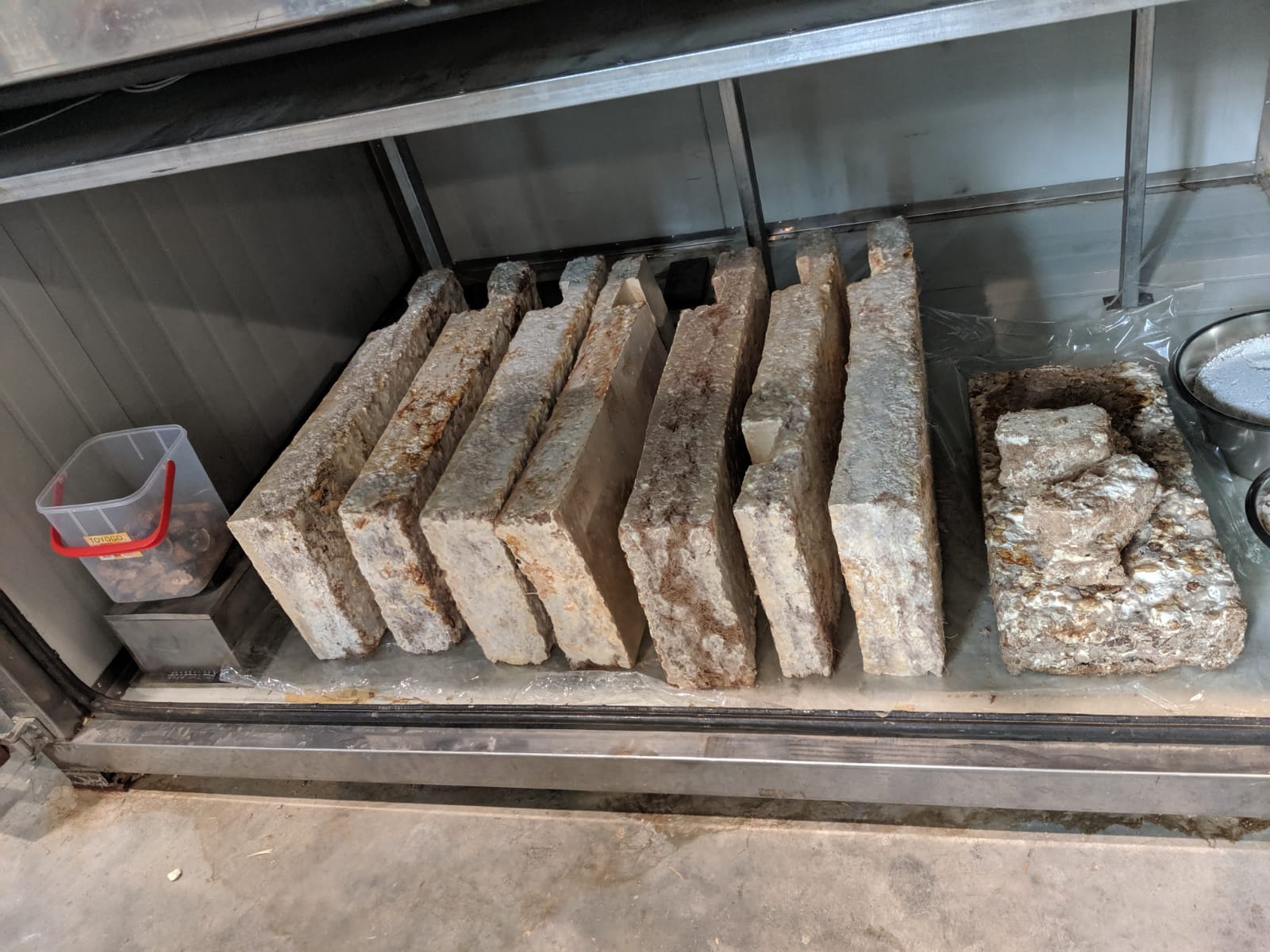


**Supplementary Fig. 3|Dried Mycelium blocks**


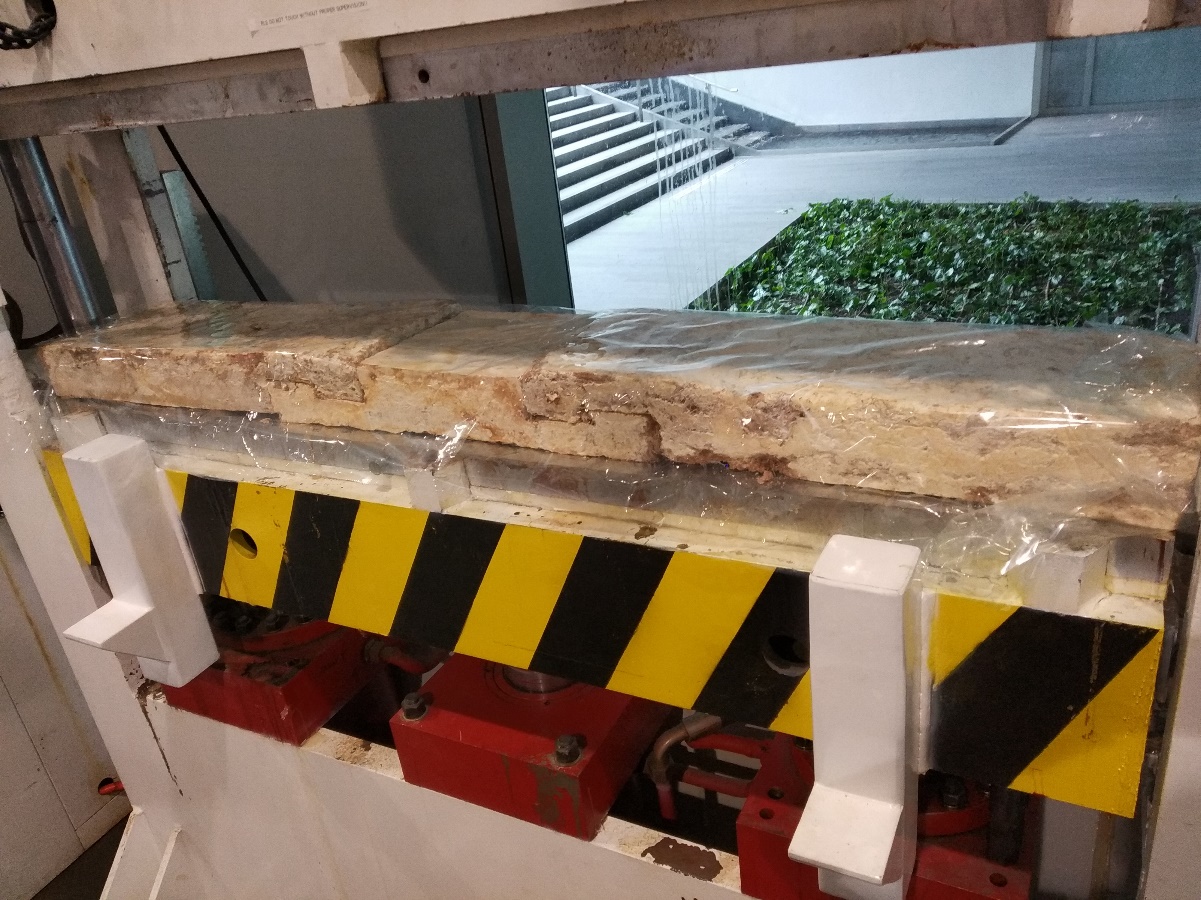


**Supplementary Fig. 4|Production of DMC under heat and pressure.**


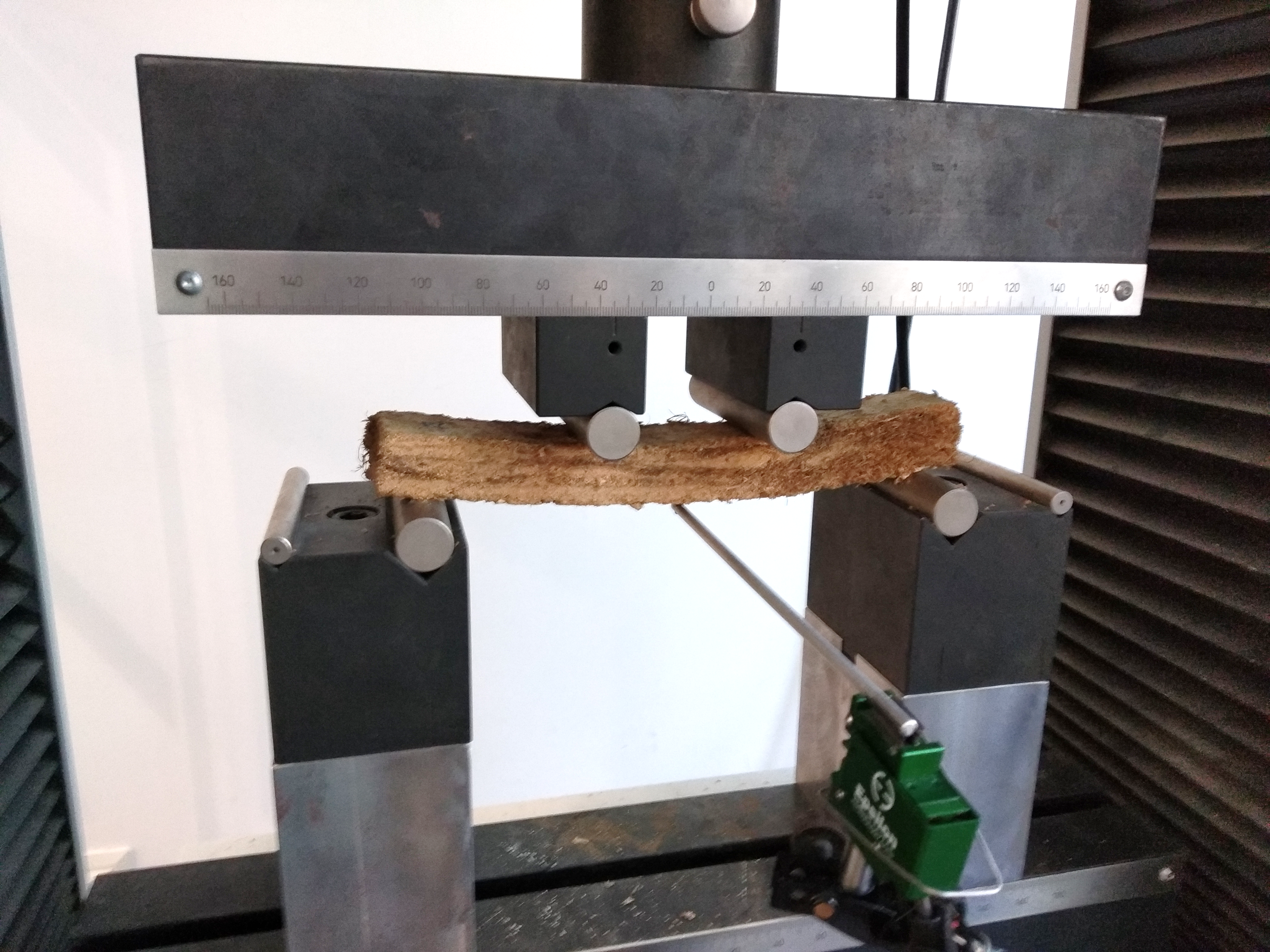


**Supplementary Fig. 5|Sample under 4-point bending test**

# Supplementary Note 2

Supplementary Tables.

**Supplementary Table 1|R_b_ raw data and ANOVA results**

| R_b_ raw data |  |  |  |  |
| --- | --- | --- | --- | --- |
|  | Day 0 | Day 7 | Day 21 | Day 35 |
| Uncoated group | 2.07 | 2.27 | 1.26 | 1.11 |
|  | 2.76 | 2.76 | 1.80 | 1.01 |
|  | 3.21 | 2.27 | 2.12 | 1.19 |
| Coated group | 1.45 | 1.74 | 1.50 | 1.22 |
|  | 2.75 | 2.71 | 2.04 | 1.85 |
|  | 3.39 | 2.53 | 2.23 | 1.79 |
| ANOVA |  |  |  |  |
| Source of Variation | df | F | P-value |  |
|  |  |  |  |  |
| Between groups | 1 | 0.30 | 0.59 |  |
|  |  |  |  |  |

**Supplementary Table 2|R_c_ raw data and ANOVA results**

| R_c_ raw data |  |  |  |  |
| --- | --- | --- | --- | --- |
|  | Day 0 | Day 7 | Day 21 | Day 35 |
| Uncoated group | 2.68 | 1.28 | 2.55 | 0.62 |
|  | 7.61 | 2.15 | 1.65 | 0.50 |
|  | 3.04 | 3.33 | 1.66 | 1.01 |
| Coated group | 2.96 | 1.31 | 1.52 | 1.49 |
|  | 4.89 | 2.94 | 2.26 | 3.59 |
|  | 2.24 | 2.06 | 2.38 | 1.55 |
| ANOVA |  |  |  |  |
| Source of Variation | df | F | P-value |  |
| Between groups | 1 | 0.03 | 0.86 |  |

**Supplementary Table 3|R_t_ raw data and ANOVA results**

| R_t_ raw data |  |  |  |  |
| --- | --- | --- | --- | --- |
|  | Day 0 | Day 7 | Day 21 | Day 35 |
| Uncoated group | 1.90 | 0.39 | 0.70 | 0.47 |
|  | 1.03 | 0.80 | 0.58 | 0.28 |
|  | 1.72 | 0.63 | 0.33 | 0.22 |
| Coated group | 1.35 | 0.81 | 1.00 | 0.78 |
|  | 1.91 | 1.06 | 0.92 | 0.82 |
|  | 1.33 | 0.46 | 0.68 | 0.82 |
| ANOVA |  |  |  |  |
| Source of Variation | df | F | P-value |  |
| Between groups | 1 | 5.22 | 0.04 |  |

| E_t_ raw data |  |  |  |  |
| --- | --- | --- | --- | --- |
|  | Day 0 | Day 7 | Day 21 | Day 35 |
| Uncoated group | 872.95 | 49.86 | 127.70 | 163.46 |
|  | 536.67 | 213.64 | 84.51 | 100.47 |
|  | 530.79 | 136.31 | 20.47 | 84.42 |
| Coated group | 485.11 | 293.20 | 190.96 | 281.15 |
|  | 696.95 | 521.76 | 386.14 | 388.43 |
|  | 624.64 | 40.15 | 168.42 | 188.60 |
| ANOVA |  |  |  |  |
| Source of Variation | df | F | P-value |  |
| Between groups | 1 | 4.19 | 0.06 |  |

**Supplementary Table 4|E_t_ raw data and ANOVA results**
